# Nordic physician-staffed prehospital services - organisation and preparedness for major emergency surgical procedures

**DOI:** 10.1186/s13049-025-01416-0

**Published:** 2025-05-23

**Authors:** Peter Galos, Karl Chevalley, Robert Larsen, Göran Sandström, Jyrki Tenhunen

**Affiliations:** 1 Department of Intensive Care Medicine and HEMS, Department of Surgical Sciences; Anaesthesiology and Intensive Care, Uppsala University Hospital, Uppsala University, Uppsala, Sweden; 2 Helicopter Emergency Medical Service, Department of Anaesthesia and Intensive Care Medicine, Institute of Clinical Sciences, Sahlgrenska Academy, Region Västra Götaland Region, University of Gothenburg, Gothenburg, Sweden; 3 Department of Anaesthesiology and Intensive Care, Department of Biomedical and Clinical Sciences, Linköping University Hospital, Linköping University, Linköping, Sweden; 4 Department of Anaesthesia and Intensive Care Medicine, Institute of Clinical Sciences, Sahlgrenska Academy, University of Gothenburg, Gothenburg, Sweden; 5 Department of War Studies, Swedish Defence University, Stockholm, Sweden

## Abstract

**Background:**

Prehospital physician-staffed services in the Nordic countries vary in crew structure, medical specialisation of crew and preparedness for major emergency surgical procedures. Performing emergency surgical procedures in prehospital settings requires equipment, training and clinical ability. This study aimed to explore the organisation of Nordic prehospital physician-staffed services and their preparedness for resuscitative thoracotomy, perimortem caesarean section and prehospital amputation.

**Methods:**

A cross-sectional survey was conducted among Nordic prehospital physician-staffed services. A web-based questionnaire was distributed to medical directors. The questions included local organisation, equipment, training, and the ability of the service to perform major emergency surgical procedures. The responses were analysed using descriptive statistics.

**Results:**

Out of 61 prehospital physician-staffed services, 54 responded (89% response rate). The various organisations showed variability in geographical coverage, staffing, and transportation options. Resuscitative thoracotomy had been carried out by 41% of the services, 85% had equipment for the procedure, and 48% had established local guidelines. Perimortem caesarean section had been performed by 7% of the services, 80% had equipment for the procedure, and 31% had established local guidelines. Prehospital amputations had been carried out by 35% of the services, 81% had equipment for the procedure, and 22% had established guidelines. Preparation for the procedures varied. 61% of the services carried out special training for resuscitative thoracotomy, 22% for perimortem caesarean section, and 39% for prehospital amputation.

**Conclusions:**

Prehospital physician-staffed units need to be prepared and have a strategy and guidelines for the treatment of unusual but life-threatening conditions. To perform major surgical procedures outside a hospital, guidelines, training, equipment, and experience are required. The study has demonstrated significant differences between Nordic countries and regions in how major surgical procedures outside the hospital are addressed. Many services lack standardised procedures and training. Addressing these gaps by implementing protocols and training programs may improve patient care. However, the potential benefits for a small number of patients should be weighed against the investment to have the ability to perform major surgical procedures outside the hospital.

**Supplementary Information:**

The online version contains supplementary material available at 10.1186/s13049-025-01416-0.

## Introduction

Prehospital physician-staffed (PPS) services in the Nordic countries have different configurations. A physician can work in a team with paramedics, HEMS (Helicopter Emergency Medical Service) crews, or nurses with specialist training. PPS units use helicopters, rapid response cars, ambulances, airplanes, or a combination thereof. Geographical conditions predominantly decide the choice of vehicles. Consistently, PPS teams, as described, have been associated with improved survival outcomes for critically ill or injured patients [1].

In the prehospital setting, there are rare occasions when physicians are compelled to consider major emergency surgical procedures generally reserved for intrahospital specialities. The scenarios requiring emergency surgical intervention include cardiac arrest due to traumatic cardiac tamponade, cardiac arrest in a third-trimester pregnant woman, and cases where a patient is trapped and deteriorating rapidly. The surgical interventions considered include resuscitative thoracotomy (RT), perimortem caesarean section (PMCS), and prehospital amputation (PA). Other potential life-saving procedures such as ECMO and REBOA require highly specialised equipment and are beyond the scope of this study.

Cardiac tamponade resulting from trauma may arise from either blunt or penetrating forces. Pre-arrest manifestations include tachycardia, hypotension, and dyspnoea. Diagnostic support is provided by ultrasonography, which may reveal pericardial effusion and indicative hemodynamic alterations. According to European Resuscitation Council (ERC) guidelines [2], RT performed via a clamshell or left anterolateral incision is recommended to restore circulatory functions in the event of a cardiac arrest due to cardiac tamponade. According to ERC, there are four specific prerequisites (4E):

1. Expertise: teams must be led by a highly trained and competent healthcare practitioner operating under a robust governance framework.
2. Equipment: adequate equipment to carry out and deal with the intrathoracic findings is mandatory.
3. Environment: ideally carried out in an operating theatre, not to be carried out if there is inadequate physical access to the patient, or if the receiving hospital is not easy to reach.
4. Elapsed time: the time without vital signs should be no longer than a maximum of 15 min.

If thoracotomy is not possible, ERC recommends considering ultrasound-guided pericardiocentesis to treat cardiac arrest associated with suspected traumatic or non-traumatic cardiac tamponade [2].

A systematic review [3] covering 72 articles and 10 238 cases of Emergency Department Thoracotomies (EDT) showed that penetrating thoracic trauma cases with no pulse but signs of life, had a 21.3% survival rate with EDT and 2.8% without EDT. With no pulse nor signs of life, there was an 8.3% survival rate with EDT, compared to a guideline group estimation of 0.2% without EDT.

In the case of blunt thoracic trauma, with no pulse but signs of life, the survival rate with EDT was 4.6%, compared to 0.5% without EDT. With no pulse nor signs of life, a 0.7% survival rate was seen, compared to a guideline group estimation of 0.001% without EDT.

In one study [4] consisting of 909 out-of-hospital traumatic cardiac arrests, 68/909 (7.5%) patients survived. These results were considered comparable to or better than survival after prehospital medical cardiac arrests. Furthermore, patients who underwent out-of-hospital thoracotomy after penetrating trauma and those who arrested after hypoxic insults had a higher chance of survival. Patients with hypovolemia as the primary cause of cardiac arrest rarely survived.

In a recent study [5] 601 cases of prehospital RT for TCA in London from January 1999 to December 2019 were analysed. Survival outcomes varied considerably depending on the cause of TCA. Among patients with cardiac tamponade, 22/105 (21%) survived, whereas only 8/418 (1.9%) with exsanguination survived. None of the 72 patients with combined or other pathologies survived. Furthermore, survival was time-limited, with no patients living beyond 15 min in cases of cardiac tamponade and 5 min in cases of exsanguination.

Cardiac arrest in pregnancy is rare with an incidence of approximately 1 in 30,000 late pregnancies [6]. Perimortem caesarean section (PMCS), or resuscitative hysterotomy, is the potential lifesaving surgical procedure in the case of maternal cardiac arrest.

The ERC guidelines [2] recommend that delivery within 5 min of collapse should be done with PMCS if.

1. > 20 weeks pregnant or the uterus is palpable above the level of the umbilicus and.
2. Immediate resuscitation (within 4 min) is unsuccessful.

This requires immediate readiness for PMCS, and surgical intervention should be considered at the scene of the cardiac arrest. However, there are no specific recommendations on out-of-hospital PMCS or time limits when not to perform the procedure in the ERC guidelines [2].

In a study [7] from 2017 involving all UK hospitals between 2011 and 2014, 66 cardiac arrests in pregnant women were identified. In 49/66 (74%) cases, PMCS was performed, and details about the procedure were available in 47 cases. In 25/47 (53%) cases, the PMCS was performed where the cardiac arrest occurred, with a maternal survival rate of 18/25 (72%). In 22/47 (47%) cases, the mother was moved for PMCS, and maternal survival was 8/22 (36%). The time to perform the PMCS was significantly shorter in the women who survived (3 min versus 12 min, *P* = 0.001). Data was available for 58 children, of whom 46/58 (79%) were born alive, 32/58 (55%) to surviving mothers and 14/58 (24%) to women who died. Although a rare condition, there are some case reports [8–10] with good outcomes for the child, when performing out-of-hospital PMCS.

Prehospital Amputation, PA, or field amputation involves the surgical removal of an upper or lower extremity to free entrapped patients and preserve life. This procedure typically includes anaesthesia, application of a proximal tourniquet, performing the amputation as distally as possible, and subsequent application of haemostats to major blood vessels [11]. While comprehensive studies on prehospital amputations are lacking, case reports [12, 13] have documented successful outcomes.

## Aims

The aims of this study were to:

1. Examine how Nordic prehospital services are organised in terms of medical specialisation, competence, training and extent of prehospital service.
2. Investigate the view of Nordic prehospital services on major emergency surgical procedures in terms of clinical practice, guidelines, equipment and training.

## Materials and methods

### Data collection

The prehospital services selected for inclusion were required to meet the following criteria:

1. Physician lead emergency response team.
2. Primary response missions.
3. Location in the Nordic countries (Denmark, Finland, Iceland, Norway and Sweden).

After e-mail correspondence with health regions of each country and additional information provided by air ambulance operators, 53 services that met the inclusion criteria were identified. Military helicopter services that could conduct HEMS missions were excluded because contact information for medical directors was not available to the authors.

The study was organised as a cross-sectional survey design in English using a self-administered questionnaire. The questions (Appendix) were divided into demographic and procedure-related sections. Questions were of open, closed and semi-closed character. The design of the web form and the questions were tested on selected colleagues, and changes were made based on the answers and feedback given.

An e-mail with study information and a link to the questionnaire [14] was sent to the medical directors of the included services in October 2023. The deadline to answer was the end of March 2024. Services that didn’t provide answers were e-mailed a reminder in January 2024, followed by contact attempts by phone. Supplementary questionnaires were distributed in April 2025 to eight additional bases that had been omitted during the initial mapping phase.

### Statistics

The results from the questionnaires were evaluated with descriptive statistics.

## Results

Out of 61 services, 54 (89%) responded. The distribution is demonstrated in Table 1.

**Table 1:** Prehospital physician-staffed services

Country	Bases identified*	Bases responded	Response Rate (%)	Inhabitants/base	km^2^ / base
Denmark	19	16	84	311 101	2 233
Finland	11	7	64	504 134	27 626
Iceland	1	1	100	375 318	100 250
Norway	21	21	100	260 684	17 394
Sweden	9	9	100	1 186 336	45 593
Total	61	54	89		

* Military services not included

### Staffing and specializations

All responding services had physicians specialised in Anaesthesiology and Intensive Care Medicine on duty (Table 2), while 6/54 (11%) also employed physicians specialised in Emergency Medicine. The medical teams included paramedics in 35/54 (65%) services, while nurses were present in 13/54 (24%). Other personnel types, such as HEMS crew or EMT, were reported by 9/54 (17%) services. Physicians and non-physicians had differences in their proportion of clinical out-of-hospital and intrahospital duty (Table 3). The distribution of working hours showed distinct patterns between physicians and non-physicians. Physicians generally combined their prehospital work with in-hospital duty. In contrast, non-physicians predominantly allocated their full working hours to prehospital activities.

**Table 2:** Medical staff and specialisations

Staff Specialization	Number of Services
Anaesthesiology and Intensive Care Medicine (Physician)	54 (100%)
Emergency Medicine (Physician)	6 (11%)
Paramedics	35 (65%)
Nurses	13 (24%)
Other	9 (17%)

**Table 3:** Distribution of clinical duty

Group	In-hospital (%)	Out-of-hospital (%)	Median (Out-of-hospital %)
Physicians	49 (SD: 21)	51 (SD: 20)	50
Non-physicians	17 (SD: 29)	83 (SD: 29)	100

### Transportation options

The survey identified diverse transportation choices among the responding prehospital services (Table 4). Most services reported having rapid response cars, while helicopters were used by two-thirds of the services. Pre-allocated ambulances were available in less than half of the services, while airplane options were the least common.

**Table 4:** Emergency transportation options

Service Option	Number of Services
Rotary Wing (Helicopter)	36 (67%)
Rapid Response Car	47 (87%)
Ambulance	18 (33%)
Airplane	6 (11%)

### Clinical procedures and training

One section of the survey focused on the three major emergency procedures (Table 5). RT had been performed by 22/54 (41%) of the services. Most of the services, 46/54 (85%), were equipped for RT, while 26/54 (48%) had established local guidelines. Only 4/54 (7%) of the services reported performing the PMCS, though 43/54 (80%) were equipped for it, and 17/54 (31%) had guidelines in place. PA had been carried out by 19/54 (35%) of the services. A total of 44/54 (81%) of the services were equipped for PA, while 12/54 (22%) had established guidelines. Training for these procedures was variably reported, with 33/54 (61%) of the services conducting specialised training for RT, 12/54 (22%) for PMCS, and 21/54 (39%) for PA. Some of the services used various training models and/or participated in courses using cadavers or other types of tissue training.

**Table 5:** Prehospital major emergency surgical procedures

Procedure	Performed	Equipped	Guidelines	Methods	Training	Courses
Resuscitative Thoracotomy (RT)	22 (41%)	46 (85%)	26 (48%)	23 prefer Clamshell, 1 lateral, 2 both	33 (61%)	34 (63%)
Perimortem Caesarean Section (PMCS)	4 (7%)	43 (80%)	17 (31%)	-	12 (22%)	16 (30%)
Prehospital Amputation (PA)	19 (35%)	44 (81%)	12 (22%)	-	21 (39%)	22 (41%)

### Geographical distribution

The population served per PPS service varied considerably across the included countries (Table 1). Sweden reported the highest average number of inhabitants per service, at 1,186,336, followed by Finland with 504,134 and Iceland with 375,318. Denmark and Norway demonstrated lower figures, with 311,101 and 260,684 inhabitants per service, respectively.

The geographical area per number of PPS services also showed considerable variation. Iceland had the largest area per service, with one service per 100 250 km². Sweden had the second-largest area per service with 45,593 km². Finland and Norway had 27,626 km² and 17,394 km², respectively. Denmark had the smallest area per physician-staffed service with 2,233 km².

## Discussion

The purpose of this study is twofold, partly a survey of Scandinavian services and partly their approach and preparedness for three selected major surgical emergency procedures.

When it comes to the mapping of PPS services, differences emerge between countries where, for example, Norway and Denmark have more than twice as many physician-staffed prehospital intensive care units as Sweden, even though the countries have a considerably smaller surface and population. This aligns with results from a similar study [15] 15 years ago.

The absence of PPS units could mean that some potentially life-saving treatments can’t be given outside hospitals. Studies [1, 16, 17] indicate that critically ill or injured patients had improved outcomes when treated by interprofessional teams led by physicians. PPS units are costly and both financial and medical aspects must be considered to ensure an overall balanced prehospital capacity with PPS services as a complement to ambulances and/or other emergency resources.

The proportion of clinical prehospital and intrahospital duty also differs between services and staff categories. A combined prehospital and hospital duty could be attributed to opportunities for a larger volume of clinical work, with more patients and thus the ability to maintain and develop general clinical skills. On the other hand, having the main part of the service within the respective PPS unit could be justified by the opportunity to develop the prehospital intensive care service and practice more on relevant critical procedures [18].

The difference in staffing regarding paramedics, nurses and HEMS crew members corresponds to earlier research [19]. This could be explained by the diverse systems for training and work within the ambulance healthcare systems between the countries. A more in-depth analysis of why these differences exist is beyond the scope of this study.

The fact that the PPS units are mainly staffed by anaesthesiologists and only a small number by emergency physicians may reflect the system of medical specialities in the Nordic countries. The former are specialists in anaesthesia, critical care and resuscitation and are responsible for initiating intensive care (e.g. general anaesthesia and intubation) anywhere in hospitals, including the emergency departments [20]. This is different from the Anglo-American tradition, where anaesthesiologists mainly serve in operating rooms, and the emergency physicians alone are responsible for resuscitation in the emergency departments [21].

Major prehospital emergency surgical procedures may be considered in specific cases that meet the established criteria. These cases present considerable challenges to the prehospital team. The physician must identify the correct medical condition, know the equipment and steps of the intervention and rapidly [2, 5, 7] perform the procedure in a potentially suboptimal environment.

Our study has shown that although the situations are rare, these procedures are currently being performed by prehospital services in the Nordic countries, with differences in preparedness.

Since PMCS and PA involve similar demanding conditions, complex decision-making processes and execution as RT, we suggest considering the following points [2, 5] when preparing a service in performing prehospital critical procedures such as major emergency surgical procedures:

1. Guidelines: Prehospital guidelines should be known and readily available to facilitate rapid decision-making and procedural actions.
2. Training: Teams involved in prehospital resuscitation should undergo regular training in both technical and non-technical skills.
3. Equipment: Appropriate, customised and easily accessible equipment should be available to the teams.
4. Experience: Teams responsible for on-scene resuscitation need experience in Critical Emergency Medical procedures, decision-making, and intensive care on-scene and during transport.
5. Organisational acceptance: Mandate and acceptance should be established by the medical directors of the involved surgical specialities.
6. Governance, research and development: Prehospital procedures and protocols, should be evaluated continuously.

### Limitations

Efforts were made to identify all PPS services, but some may have been inadvertently overlooked. The survey response rate varied between countries; all PPS services in Sweden, Norway, and Iceland responded, whereas the response rate from Finnish PPS services was only 64%. This discrepancy may influence the results.

Military helicopter services were excluded from this study. This is a limitation as military helicopters carry out primary missions in some Nordic countries.

There is uncertainty in coverage per area and capita; some services have not responded, and some services in larger cities have multiple teams operating in parallel.

The practice and frequency of the prehospital intensive care team collaborating with local emergency services using their ambulance for hospital transport were not examined.

## Conclusions

PPS units need to be prepared and have a strategy and guidelines for the treatment of unusual but life-threatening conditions. To perform major emergency surgical procedures outside a hospital, guidelines, training, equipment, experience are required.

As these patients are uncommon and the treatment involves emergency surgical procedures outside its normal context, we believe it is important that the healthcare system has acceptance, governance and support from the specialists in the receiving hospitals. Major surgical emergency prehospital procedures require research and continuous evaluation.

The study has demonstrated significant differences between Nordic countries and regions in how major surgical procedures outside the hospital are addressed. Many services lack standardised procedures and training. Addressing these gaps by implementing protocols and training programs may improve patient care.

The potential benefits for a small number of patients should be weighed against the investment to have the ability to perform major surgical procedures outside the hospital.

## Electronic supplementary material

Below is the link to the electronic supplementary material.

Supplementary Material 1

## Data Availability

No datasets were generated or analysed during the current study.
