# Supplementary material for: Nordic physician-staffed prehospital services - organisation and preparedness for major emergency surgical procedures
Source: Scand J Trauma Resusc Emerg Med. 2025 May 23;33:95. doi: 10.1186/s13049-025-01416-0 (PMC12103025; doi:10.1186/s13049-025-01416-0)
Supplement: Supplementary file 1 — Supplementary Material 1 [file 13049_2025_1416_MOESM1_ESM.docx]

# Appendix: Questionary

| **Question** | **Type of Answer Options** |
| --- | --- |
| **Country of your service** | Closed end (multiple choice) |
| **Where is your service located?** | Open end |
| **Vehicle options of your service** | Closed end (Yes/No) |
| **Team setup** | Semi-closed (multiple choice with an additional text field) |
| **Specialisation of Physician** | Semi-closed (multiple choice with an additional text field) |
| **Estimated percentage of service being out of hospital and working in the hospital** | Open end (numerical input) |
| **Specialisation of Nurse/Paramedic/...** | Semi-closed (multiple choice with an additional text field) |
| **Prehospital Resuscitative thoracotomy** | Closed end (Yes/No) with additional text fields for comments and specifications |
| **If guidelines exist which approach is planned for Resuscitative thoracotomy?** | Semi-closed (multiple choice with an additional text field) |
| **Has Prehospital Resuscitative thoracotomy been performed at your service?** | Closed end (frequency options) |
| **Comments regarding prehospital resuscitative thoracotomy** | Open end |
| **Prehospital Perimortem caesarean section (resuscitative hysterotomy)** | Closed end (Yes/No) with additional text fields for comments and specifications |
| **Has Prehospital resuscitative hysterotomy been performed at your service?** | Closed end (frequency options) |
| **Comments regarding prehospital resuscitative hysterotomy** | Open end |
| **Prehospital field amputation** | Closed end (Yes/No) with additional text fields for comments and specifications |
| **Has Prehospital field amputation been performed at your service?** | Closed end (frequency options) |
| **Comments regarding prehospital field amputation** | Open end |
| **If you have guidelines for prehospital Resuscitative thoracotomy, Resuscitative hysterotomy or field amputation, please attach them here** | Open end (file upload) |
